# Factors affecting number of lymph nodes harvested and the impact of examining a minimum of 12 lymph nodes in stage I-III colorectal cancer patients: a retrospective single institution cohort study of 1167 consecutive patients

**DOI:** 10.1186/s12893-016-0132-7

**Published:** 2016-04-14

**Authors:** Hsiang-Lin Tsai, Ching-Wen Huang, Yung-Sung Yeh, Cheng-Jen Ma, Chao-Wen Chen, Chien-Yu Lu, Ming-Yii Huang, I-Ping Yang, Jaw-Yuan Wang

**Affiliations:** Division of Gastroenterology and General Surgery, Department of Surgery, Kaohsiung Medical University Hospital, Kaohsiung Medical University, Kaohsiung, Taiwan; Graduate Institute of Medicine, College of Medicine, Kaohsiung Medical University, Kaohsiung, Taiwan; Department of Surgery, Faculty of Medicine, College of Medicine, Kaohsiung Medical University, Kaohsiung, Taiwan; Division of Colorectal Surgery, Department of Surgery, KaohsiungMedical University Hospital, Kaohsiung, Taiwan; Graduate Institute of Clinical Medicine, College of Medicine, Kaohsiung Medical University, Kaohsiung, Taiwan; Division of Trauma, Department of Surgery, Kaohsiung Medical University Hospital, Kaohsiung Medical University, Kaohsiung, Taiwan; Department of Emergency Medicine, Faculty of Medicine, College of Medicine, Kaohsiung Medical University, Kaohsiung, Taiwan; Division of Gastroenterology, Department of Internal Medicine, Kaohsiung Medical University Hospital, Kaohsiung Medical University, Kaohsiung, Taiwan; Department of Internal Medicine, Faculty of Medicine, College of Medicine, Kaohsiung Medical University, Kaohsiung, Taiwan; Department of Radiation Oncology, Kaohsiung Medical University Hospital, Kaohsiung Medical University, Kaohsiung, Taiwan; Department of Radiation Oncology, Faculty of Medicine, College of Medicine, Kaohsiung Medical University, Kaohsiung, Taiwan; Department of Nursing, Shu-Zen College of Medicine and Management, Kaohsiung, Taiwan; Center for Biomarkers and Biotech Drugs, Kaohsiung Medical University, Kaohsiung, Taiwan

**Keywords:** Lymph nodes harvested, Colorectal cancer, Disease-free survival, Overall survival, Impact of examining lymph nodes

## Abstract

**Background:**

To identify factors affecting the harvest of lymph nodes (LNs) and to investigate the association between examining a minimum of 12 LNs and clinical outcomes in stage I-III colorectal cancer (CRC) patients.

**Methods:**

The clinicopathologic features and the number of examined LNs for 1167 stage I-III CRC patients were analyzed to identify factors affecting the number of LNs harvested and the correlations between clinical outcomes and high harvests (≧12 LNs) and low harvests (<12 LNs).

**Results:**

A multivariate analysis showed that age (*P* = 0.007), tumor size (*P* = 0.030), and higher T stage (*P* = 0.001) were independent factors affecting the examinations of LNs in colon cancer and that tumor size (*P* = 0.015) was the only independent factor in rectal cancer. Patients with low harvests had poorer overall survival with stage II and stage III CRC (stage II: *P* < 0.0001; III: *P* = 0.001) and poorer disease-free survival for stages I-III (stage I: *P* = 0.023; II: *P* < 0.0001; III: *P* = 0.001).

**Conclusions:**

The factors influencing nodal harvest are multifactorial, and an adequate number of examined LNs (≧12) is associated with a survival benefit. Removal of at least 12 LNs will determine the lymph node status reliably.

## Background

In the 7th edition of the AJCC Cancer Staging Handbook published by the American Joint Committee on Cancer (AJCC) and the International Union Against Cancer (UICC), the recommendation for the number of lymph nodes (LNs) that need to be removed is 10–14 [1]. As the detection of positive LNs is critical for the prediction of patient outcomes, an adequate number of LNs must be examined. In a commentary on TNM classification, Compton and Green indicated that at least 12 LNs need to be removed [2]. In addition, the “College of American Pathologists Consensus Statement” [3] and the clinical practice guidelines from the National Comprehensive Cancer Network [4] also recommend that at least 12 LNs be removed.

Despite emerging data on the prognostic utility of molecular profiling, pathologic assessment of resected specimens remains the strongest predictor of survival in patients with CRC. Goldstein et al. [5] used mathematical models to show a direct linear relationship between LN yield and the probability of finding a positive node. Moreover, LN harvest has been shown to impact clinical outcomes in patients with stage II and stage III disease, such that more negative LNs confer a survival advantage [6–8]. In addition, Sarli et al. reported that the factors influencing nodal harvest are multifactorial [9]. Three key variables are thought to impact LN yield, including patient-dependent factors, surgeon-dependent factors, and the pathologic assessment of specimens.

Data from large series of cases from single institutions are rare. The aims of this retrospective study were to address this issue by identifying the factors affecting the harvest of LNs and by examining the associations between the 12-LN measure and clinical outcomes to analyze the impact of the pathological examination of 12 regional LNs on overall survival (OS) and disease-free survival (DFS) in patients with stage I-III CRC following radical resection.

## Methods

### Patient selection

This was a retrospective single institution cohort study. Between January 2006 and October 2013, a total of 1340 elective and consecutive patients with stage I-III CRC, as defined by the Union for International Cancer Control (UICC), who underwent radical resection at a single institution were considered for enrollment, with those patients who experienced surgery-related death (66 patients), who were lost to follow-up for over one year (86 patients), or who received neoadjuvant treatment (21 patients) being excluded. Ultimately, we enrolled 1167 stage I-III CRC patients. All the enrolled patients received detailed studies, including laboratory data analyses, colonofiberscopy, image studies (i.e., abdominal computed tomography (CT), chest X-ray, magnetic resonance imaging (MRI), etc.) before surgery. All clinical data were obtained with informed consent from each subject, and the study protocol was approved by the Institutional Review Board of Kaohsiung Medical University Hospital (KMUHIRB-E-20150004).

The resected specimen from each patient was fixed in 10 % formalin solution and then routinely processed for paraffin embedding. Conventional methods of visual inspection and palpation were used to identify the LNs. The histological type (adenocarcinoma or mucinous carcinoma if > 50 % of the tumor volume was composed of mucin) and histological grade of each tumor specimen were evaluated. Development of a new local recurrence (tumor growth restricted to the anastomosis or the region of the primary operation) or distant metastatic lesions (distant metastases or diffuse peritoneal carcinomatosis) during the period of postoperative surveillance was defined as a postoperative relapse [10]. All enrolled patients were followed up until death or December 2014.

### Type of surgery

The patients made their own choices with regard to receiving open surgery or laparoscopy-assisted surgery. According to the tumor location, we carried out whichever of the two surgical methods was chosen in accordance with the standard procedure for the given method. Radical (R0) resection is defined as any gross residual tumor that did not remain in the surgical bed, and the surgical resection margin is pathologically negative for tumor invasion. Total mesorectal excision was performed in all patients with tumors of the middle and lower rectum and a distal clearance of at least 2 cm from the edge of the tumor.

### Detection of serum CEA, vascular invasion, and perineural invasion

A 3-ml peripheral blood sample was obtained from each of the 1167 CRC patients less than 1 week prior to the operation (preoperative CEA). Serum CEA levels were also determined by means of an enzyme immunoassay test kit (Beckman Coulter, Inc., Fullerton, CA), with an upper limit of 5 ng/ml being defined as normal according to the manufacturer of the kits that were used. Vascular invasion was identified on the basis of one or more of the following: tumor cells lining the venous endothelial surface, tumor cell thrombi inside the lumen of the vein, or destruction of the vein wall by tumor cells. Perineural invasion was identified when a positive judgment was made when cancer cells were observed extraneurally.

### Definition of regional lymph nodes

Pericolic lymph nodes and nodes along the trunks of named vessels are defined by the International Union Against Cancer (UICC) as regional lymph nodes (ileocolic, right colic, middle colic, superior mesenteric, left colic, inferior mesenteric, and sigmoidal arteries). Metastases in all other nonregional lymph nodes (e.g., interaortocaval, external iliac) are regarded as distant metastases [11, 12].

### Clinicopathological features and postoperative surveillance

The clinicopathological features analyzed in this investigation included the patients’ gender, age, tumor size, tumor location, UICC stage, depth of invasion, numbers of examined lymph nodes, vascular invasion, perineural invasion, tumor grade, tumor histology, preoperative carcinoembryonic antigen (CEA) level and type of surgery. Adjuvant chemotherapy was administrated to patients with high-risk stage II and stage III CRC according to the treatment guidelines of our institution. The high-risk stage II CRC patients included those with colonic obstruction or perforation, T4 invasive depth, positive vascular invasion, numbers of lymph node retrieval less than 12, and poorly differentiated adenocarcinoma. Postoperative surveillance consisted of a medical history, physical examination, and laboratory studies, including serum CEA levels every 3 months. Abdominal ultrasonography was performed every 6 months, and chest radiography and abdominal or chest CT scans were performed once a year or as each patient’s clinical condition indicated. The enrolled patients were followed up at 3-month intervals for an initial 2 years and then at 6-month intervals thereafter till 5 years.

### Disease-free survival (DFS), overall survival (OS) and TNM stage

We estimated the correlations between disease-free survival (DFS), overall survival (OS), and the different UICC stages according to the adequacy of the number of lymph nodes retrieved. DFS was defined as the length of time after primary surgery during which a patient survives with no sign of CRC. OS was defined as the time elapsed between the primary surgery and death from any cause.

### Statistical analysis

Continuous variables are presented as mean ± standard deviation (SD), and dichotomous variables are presented as number and percentage values. All statistical analyses were performed using the Statistical Package for the Social Sciences, version 19.0 (SPSS, Inc., Chicago, IL). According to the number of LNs harvested, patients were categorized into a high-harvest group (≧12 LNs examined) or low-harvest group (<12 LNs examined). The clinicopathological characteristics of these two groups were compared using either the Pearson chi-square or ANOVA test, as appropriate. Binary logistic regression was used to evaluate the association between the 12-lymph node measure and clinicopathological variables found to be significant in univariate analyses. Logistic regression coefficients were used to estimate odds ratios (OR) for each of the independent variables in the model. Patient survival was estimated by the Kaplan-Meier method, and the log rank test was used to determine the difference. A *P* value of less than 0.05 was considered statistically significant.

## Results

### Demographics of the enrolled 1167 stage I-III CRC patients

**Fig. 1:**
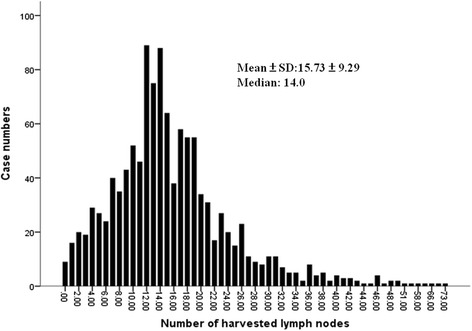
The distribution of examined lymph nodes from 1167 stage I-III colorectal cancer patients who underwent radical resection

Demographic data for the enrolled 1167 patients are shown in Table 1. We followed the enrolled 1167 patients until December 2014, with a mean follow-up period of 44.17 ± 26.13 months (range: 12–107 months). We further found that 807 patients (69.2 %) were in the high-harvest group; meanwhile, 360 patients (30.8 %) were in the low-harvest group. Figure 1 shows the distribution of the case numbers; the mean value ± SD and median value were 15.73 ± 9.29 and 14, respectively.

**Table 1:** The clinicopathologic characteristics of 1167 stage I-III colorectal cancer patients following radical resection

Variables	Number (%)
Gender	
Male/Female	691 (59.2)/476 (40.8)
Age (y/o)	
≧65/<65	610 (52.3)/557 (47.7)
Maximum size (cm)	
≧5/<5	414 (35.5)/753 (64.5)
Tumor location	
Colon/Rectum	797 (68.3)/370 (31.7)
Tumor stage	
I/II/III	302 (25.9)/425 (36.4)/440 (37.7)
T stage	
T1/T2/T3/T4	121 (10.4)/239 (20.5)/723 (61.9)/84 (7.2)
Harvested LN No^a^	
≧12/<12	807 (69.2)/360 (30.8)
Vascular invasion	
Yes/No	261 (22.4)/906 (77.6)
Perineural invasion	
Yes/No	303 (26.0)/864 (74.0)
Tumor grade	
WD/MD/PD^b^	96 (8.3)/969 (83.0)/102 (8.7)
Tumor histology	
A/M/S^c^	1097 (94.0)/66 (5.7)/4 (0.3)
Preoperative CEA^d^ (ng/ml)	
≧5/<5	441 (35.2)/756 (64.8)
Type of surgery	
Open/Laparoscopic	967 (82.9)/200 (17.1)

^a^ *LN No* numbers of lymph nodes

^b^ *WD* well differentiated, *MD* moderately differentiated, *PD* poorly differentiated

^c^ *A* adenocarcinoma, *M* mucinous carcinoma, *S* signet-ring cell carcinoma

^d^ *CEA* carcinoembryonic antigen

### Association between clinicopathologic features and the number of examined LNs

#### For colon cancer patients

Based on a univariate analysis of the correlations between the examined number of LNs (high-harvest group vs. low-harvest group) and clinicopathologic features, we found that there were statistically significant differences between the two groups in terms of age (*P* = 0.004), tumor size (*P* = 0.001), depth of tumor invasion (*P* < 0.0001), and TNM stage (*P* = 0.001) (Table 2). However, there were no significant differences in terms of gender, tumor histology, tumor grade, vascular invasion, perineural invasion, type of surgery, and preoperative CEA level. Using a multivariate logistic regression analysis, we found that the high-harvest group was statistically younger than the low-harvest group (*P* = 0.007; odds ratio [OR], 1.582; 95 % confidence interval [CI], 1.131–2.212), had a larger mean size of tumor (*P* = 0.030; OR, 1.503; 95 % CI, 1.040–2.171), and had a more advanced mean depth of invasion (*P* = 0.001; OR, 1.919; 95 % CI, 1.318–2.795).

**Table 2:** Univariate analysis and logistic regression analysis correlations between the number of harvested lymph nodes (≧12 LNs examined in the high-harvest group or < 12 LNs examined in the low-harvest group) and other variables among 797 stage I-III colon cancer patients

	Low-harvest group	High-harvest group	Univariate analysis	Logistic regression analysis
Variables	(*n* = 197) (%)	(*n* = 600) (%)	*P*-value	OR^a^ (95 % CI^b^) *P*-value
Gender				
Male/Female	115 (58.4)/82 (41.6)	339 (56.5)/261 (43.5)	0.645	- -
Age (y/o)				
≧65/<65	120 (60.9)/77 (39.1)	295 (49.2)/305 (50.8)	0.004	1.582 (1.131–2.212) 0.007
Tumor size (cm)				
< 5/≧5	140 (71.1)/57 (28.9)	347 (57.8)/253 (42.2)	0.001	1.503 (1.040–2.171) 0.030
Depth of invasion				
T1 + T2/T3 + T4	81 (41.2)/116 (58.8)	134 (22.4)/466 (77.6)	<0.0001	1.919 (1.318–2.795) 0.001
TNM stage				
I + II/III	141 (71.6)/56 (28.4)	350 (58.3)/250 (41.7)	0.001	1.427 (0.984–2.070) 0.061
Tumor histology				
M + S/A^c^	13 (6.6)/184 (93.4)	39 (6.5)/561 (93.5)	0.961	- -
Tumor grade				
WD + MD/PD^d^	181 (91.8)/16 (8.2)	539 (89.8)/61 (10.2)	0.399	- -
Vascular invasion				
Yes/No	46 (23.4)/151 (76.6)	131 (21.8)/469 (78.2)	0.657	- -
Perineural invasion				
Yes/No	49 (24.9)/148 (75.1)	146 (24.4)/454 (75.6)	0.878	- -
Type of surgery				
Laparoscopic/Open	38 (19.3)/159 (80.7)	101 (16.8)/499 (83.2)	0.431	- -
Preoperative CEA^e^ (ng/ml)				
≧5/<5	68 (34.5)/129 (65.5)	217 (36.2)/383 (63.8)	0.675	- -

^a^ *OR* odds ratio

^b^95 % CI 95 % confidence interval

^c^ *M* mucinous carcinoma, *S* signet-ring cell carcinoma, *A* adenocarcinoma

^d^ *PD* poorly differentiated, *MD* moderately differentiated, *WD* well differentiated

^e^ *CEA* carcinoembryonic antigen

#### For rectal cancer patients

Table 3 shows the correlations between clinicopathologic features and harvested lymph nodes for 370 UICC stage I-III rectal cancer patients. Using univariate analysis, it was demonstrated that there were statistically significant differences between the two groups in terms of tumor size (*P* = 0.006) and TMN stage (*P* = 0.023). However, there were no significant differences with regard to the remaining variables. Furthermore, we used a multivariate logistic regression analysis and found only that the mean size of tumor (*P* = 0.015; OR, 1.855; 95 % CI, 1.127–3.053) was significantly larger for the high-harvest group than for the low-harvest group.

**Table 3:** Univariate analysis and logistic regression analysis correlations between the number of harvested lymph nodes (≧12 LNs examined in the high-harvest group or < 12 LNs examined in the low-harvest group) and other variables among 370 stage I-III rectal cancer patients

	Low-harvest group	High-harvest group	Univariate analysis	Logistic regression analysis
Variables	(*n* = 163) (%)	(*n* = 207) (%)	*P*-value	OR^a^ (95 % CI^b^) *P*-value
Gender				
Male/Female	103 (63.2)/60 (36.8)	134 (64.7)/73 (35.3)	0.759	- -
Age (y/o)				
≧65/<65	120 (60.9)/77 (39.1)	295 (49.2)/305 (50.8)	0.285	- -
Tumor size (cm)				
< 5/≧5	129 (79.1)/34 (20.9)	137 (66.2)/70 (33.8)	0.006	1.855 (1.127–3.053) 0.015
Depth of invasion				
T1 + T2/T3 + T4	81 (41.2)/116 (58.8)	134 (22.4)/466 (77.6)	0.189	0.945 (0.588–1.521) 0.817
TNM stage				
I + II/III	114 (69.9)/49 (30.1)	121 (58.4)/86 (41.6)	0.023	1.499 (0.925–2.431) 0.101
Tumor histology				
M + S/A^c^	8 (4.9)/155 (95.1)	10 (4.8)/197 (95.2)	0.973	- -
Tumor grade				
WD + MD/PD^d^	153 (93.8)/10 (6.2)	193 (93.2)/14 (6.8)	0.808	- -
Vascular invasion				
Yes/No	31 (19.1)/132 (80.9)	53 (25.6)/154 (74.4)	0.133	1.210 (0.702–2.087) 0.492
Perineural invasion				
Yes/No	45 (27.7)/118 (72.3)	63 (3054)/144 (69.5)	0.553	- -
Type of surgery				
Laparoscopic/Open	28 (17.2)/135 (82.8)	33 (15.9)/174 (84.1)	0.750	- -
Preoperative CEA^e^ (ng/ml)				
≧5/<5	54 (33.2)/109 (66.8)	72 (34.8)/135 (65.2)	0.739	- -

^a^ *OR* odds ratio

^b^ *95 % CI* 95 % confidence interval

^c^ *M* mucinous carcinoma, *S* signet-ring cell carcinoma, *A* adenocarcinoma

^d^ *PD* poorly differentiated, *MD* moderately differentiated, *WD* well differentiated

^e^ *CEA* carcinoembryonic antigen

### Disease-free survival and overall survival based on the number of examined LNs

**Fig. 2:**
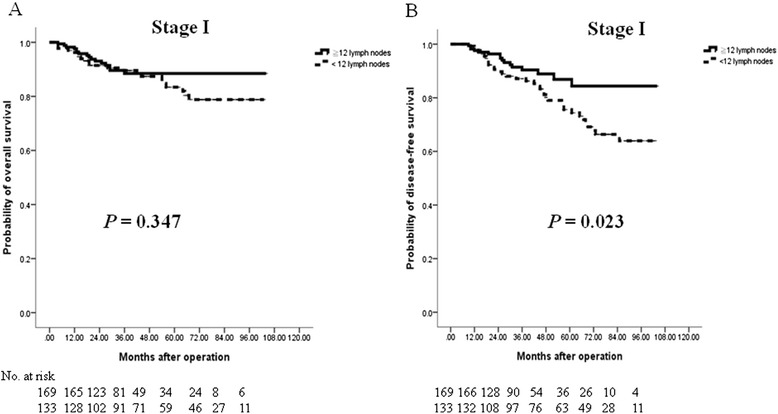
Cumulative survival rates of the 302 enrolled patients with stage I colorectal cancer (CRC) who underwent curative resection as assessed by the Kaplan-Meier method. The differences in survival rates were analyzed by the log-rank test. **a** The rates of overall survival were not significantly different between the high-harvest group (≧12 LNs examined) and the low-harvest group (<12 LNs examined) in stage I CRC patients. **b** The disease-free survival was significantly lower in the low-harvest group than in the high-harvest group

**Fig. 3:**
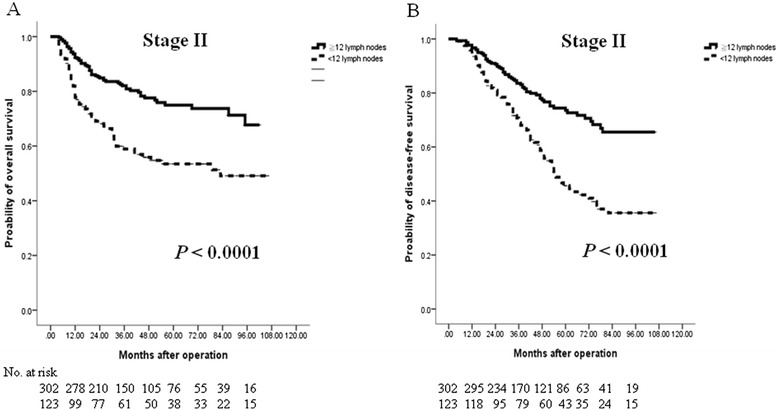
Cumulative survival rates of the 425 enrolled patients with stage II colorectal cancer (CRC) who underwent curative resection as assessed by the Kaplan-Meier method. The differences in survival rates were analyzed by the log-rank test. **a** The overall survival was significantly lower in the low-harvest group (<12 LNs examined) than in the high-harvest group (≧12 LNs examined). **b** The disease-free survival was also considerably lower in the low-harvest group than in the high-harvest group

**Fig. 4:**
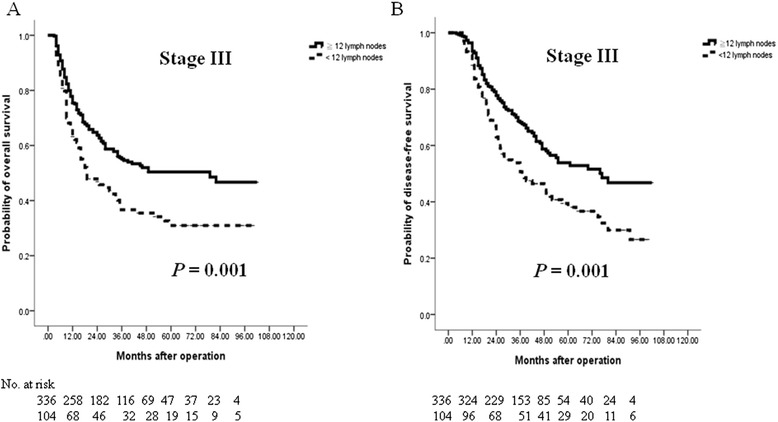
Cumulative survival rates of the 440 enrolled patients with stage III colorectal cancer (CRC) who underwent curative resection as assessed by the Kaplan-Meier method. The differences in survival rates were analyzed by the log-rank test. **a** The overall survival was significantly lower in the low-harvest group (<12 LNs examined) than in the high-harvest group (≧12 LNs examined). **b** The disease-free survival was also considerably lower in the low-harvest group than in the high-harvest group

We further validated the clinical significance of the number of examined LNs on DFS and OS in stage I-III CRC patients. For stage I cases, the OS of CRC patients in the low-harvest group was not significantly lower than that of the CRC patients in the high-harvest group (*P* = 0.347; Fig. 2a); however, the DFS was significantly lower in the low-harvest group (*P* = 0.023; Fig. 2b). For stage II cases, both the DFS and OS were significantly higher in the CRC patients in the high-harvest group (both *P* < 0.0001; Fig. 3). Likewise, the results also indicated better outcomes for stage III CRC patients in the high-harvest group (both *P* = 0.001; Fig. 4).

## Discussion

The novel aspects and findings of the present study were as follows: (1) there appeared to be only one independent factor affecting the harvest of lymph nodes for stage I–III rectal cancer patients; (2) we found that an adequate number of retrieved LNs was most significantly associated with the DFS of stage I-III CRC patients and the OS of stage II and III CRC patients; and (3) this is the first comprehensive report identifying significant factors predicting the retrieval of lymph nodes in colon cancer and rectal cancer respectively.

Previous researchers have demonstrated a relationship between the number of histologically examined lymph nodes and prognosis: the more LNs examined, the better the prognosis [13–16]. Numerous observational studies have also shown that CRC patients for whom an adequate number of LNs have been examined have a considerably lower rate of mortality [17, 18]. The 12 lymph node benchmark for LN retrieval and examination arose in recent years from the observed relationship between higher numbers of LNs and improved patient survival [19]. Moreover, the results of this study support previous findings that there are many factors influencing the harvest of regional lymph nodes [9].

Our results are similar to the finding of previous studies that the number of LNs harvested is inversely associated with the age of colon cancer patients [20, 21]. Inflammation and its contributing process have been known to cause LNs to become enlarged, making them easier to identify than normal nodes among both surgeons and pathologists [22]. Tekkis et al. [23] reported that older individuals could have reduced immunologic and inflammatory reactions to cancers that would lead to decreased numbers of LNs being harvested. It is also possible that LNs may undergo a process of involution with increasing age.

One interesting finding of the current study is that larger tumors are more likely to result in a higher nodal harvest regardless of whether the given patient has colon cancer or rectal cancer, a result which is consistent with the observations of Chen et al. [24]. A biological explanation for this finding is not yet known, but it is possible that larger tumors may lead to more profound inflammatory responses in the mesentery basin, resulting in a greater number of LNs (though not necessarily positive LNs) in the resected mesentery being harvested for pathological examination. In addition, it is noteworthy that our data indicated that T-descriptor, which refers to the depth of wall penetration in the colon was directly correlated with the number of LNs retrieved. A deeper penetration of the bowel wall found in patients with T3 or T4 tumors may result in a greater antigenic immune and inflammatory response within the regional LNs, making them more apparent to pathologic examination [20]. In a population-based study of 2281 CRC patients, Kelder et al. [25] also demonstrated that T stage was independently associated with the number of examined LNs. In that study, the number of LNs recovered increased with advanced TNM stage, and we found the controversial results in our study.

In this study, the mean examined number of LNs was 15.73 ± 9.29. Our enrolled patients with LNs examination are adherent to the LN quality measure, of which the 12 LNs goal is nearly 70 %. Three population-based studies have confirmed the impact of a high number of examined LNs (≧12) on long-term survival in patients with stage II CRC [26, 27]. However, some reports on stage III CRC are contradictory. Vather et al. reported that the 5-year survival rate was increased with increased nodal harvests, with the greatest difference occurring at the < 12 and ≧12 LNs cut-off [27]. Conversely, Kelder et al. reported no significant difference in survival in three subsets of nodal harvests (<6, 6–11, and ≧12) in 738 stage III patients [25]. We found that stage I-III CRC patients with a higher number of examined LNs (≧12) had better overall survival rates than those with a lower number of examined LNs (<12). In terms of DFS, the same observations applied except for among the stage I CRC patients. This may have been due to the relatively low rate of recurrence for the stage I CRC patients. Herein, we propose three possible mechanisms for the detrimental impact of low LN harvests on survival: (1) surgeon-related factors, with worse survival being associated with less complete resections, which also lead to less nodes being removed [28]; (2) pathologist-related factors, with worse survival occurring due to the phenomenon of stage migration [29]; (3) a mechanism reflecting interactions between the tumor and the host that may also be associated with prognosis [30].

The limitations of this study were as follows: (1) it consisted of a retrospective cohort study involving only a single institute; (2) it involved many surgeons, and surgeon-dependent factors might be among the most important factors affecting the harvest of lymph nodes; and (3) we did not categorize the surgeries by the anatomic type of resection (e.g. right-hemicolectomy, left-hemicolectomy, sigmoidectomy, extended, lower anterior resection etc.), which may have resulted in a statistical bias. More accurate identification of patients with an adequate retrieval of lymph nodes and appropriate intensive treatment/follow-up, especially systematic chemotherapy, may improve the efficacy of various treatments and overall survival. However, it will be necessary to analyze clinical data from multiple institutions in order to develop simpler, more sensitive, and specific criteria for detecting patients with a high prediction of sufficient lymph-node retrieval and, in turn, to improve clinical outcomes.

## Conclusion

This study further confirmed that a total of 12 or more examined LNs is associated with increased long-term survival in stage I-III CRC patients. Furthermore, a minimum of 12 LNs examined is not only the standard of care, and thus important in predicting outcomes and directing treatment goals, but is also associated with a reduction in mortality.

## References

[1] Edge SB, Compton CC (2010). The American Joint Committee on Cancer: the 7th edition of the AJCC cancer staging manual and the future of TNM. Ann Surg Oncol.

[2] Compton CC, Green FI (2004). The staging of colorectal cancer: 2004 and beyond. CA Cancer J Clin.

[3] Compton CC, Fielding LP, Burgart LJ, Conley B, Cooper HS, Hamilton SR (2000). Prognostic factors in colorectal cancer. College of American Pathologists Consensus Statement 1999. Arch Pathol Lab Med.

[4] National National Comprehensive Cancer Network, NCCN Clinical Practice Guidelines in Oncology: Colon Cancer, Rectal Cancer, Version 1. 2014, NCCN, 2014. httt://www.nccn.org/professionals. 10.6004/jnccn.2009.0056 19755046

[5] Goldstein NS, Sanford W, Coffey M, Layfield LJ (1996). Lymph node recovery from colorectal resection specimens removed for adenocarcinoma. Trends over time and a recommendation for a minimum number of lymph nodes to be recovered. Am J Clin Pathol.

[6] Swanson RS, Compton CC, Stewart AK, Bland KI (2003). The prognosis of T3N0 colon cancer is dependent on the number of lymph nodes examined. Ann Surg Oncol.

[7] Johnson PM, Porter GA, Ricciardi R, Baxter NN (2006). Increasing negative lymph node count is independently associated with improved long-term survival in stage IIIB and IIIC colon cancer. J Clin Oncol.

[8] Tsai HL, Yeh YS, Yu FJ, Lu CY, Chen CF, Chen CW (2009). Predicting factors of postoperative relapse in T_2-4_N_0_M_0_ colorectal cancer patients via harvesting a minimum of 12 lymph nodes. Int J Colorectal Dis.

[9] Sarli L, Bader G, Iusco D, Salvemini C, Mauro DD, Mazzeo A (2005). Number of lymph nodes examined and prognosis of TNM stage II colorectal cancer. Eur J Cancer.

[10] Tsai HL, Yang IP, Lin CH, Chai CY, Huang YH, Chen CF (2013). Predictive value of vascular endothelial growth factor overexpression in early relapse of colorectal cancer patients after curative resection. Int J Colorectal Dis.

[11] Hogan NM, Winter DC (2013). A nodal positivity constant: new perspectives in lymph node evaluation and colorectal cancer. World J Surg.

[12] Hida J, Okuno K, Yasutomi M, Yoshifuji T, Matsuzaki T, Uchida T (2005). Number versus distribution in classifying regional lymph node metastases from colon cancer. J Am Coll Surg.

[13] Tsai HL, Lu CH, Hsieh JS, Wu DC, Jan CM, Chai CY (2007). The prognostic significance of total lymph node harvest in patients with T2-4N0M0 colorectal cancer. J Gastrointest Surg.

[14] Rosenberg R, Engel J, Bruns C, Heitland W, Hermes N, Jauch KW (2010). The prognostic value of lymph node ratio in a population-based collective of colorectal cancer patients. Ann Surg.

[15] Kotake K, Honjo S, Sugihara K, Hashiguchi Y, Kato T, Kodaira S (2012). Number of lymph nodes retrieval is an important determinant of survival of patients with stage II and stage III colorectal cancer. Jpn J Clin Oncol.

[16] Sjo OH, Merok MA, Svindland A, Nesbakken A (2012). Prognostic impact of lymph node harvest and lymph node ratio in patients with colon cancer. Dis Colon Rectum.

[17] Chen SL, Bilchik AJ (2006). More extensive nodal dissection improves survival for stage I to III of colon cancer: a population-based study. Ann Surg.

[18] Bui L, Rempel E, Reeson D, Simunovic M (2006). Lymph node counts, rates of positive lymph nodes, and patient survival for colon cancer surgery in Ontario, Canada: A population-based study. J Surg Oncol.

[19] Chang GJ, Rodriguez-Bigas MA, Skibber JM, Moyer VA (2007). Lymph node evaluation and survival after curative resection of colon cancer: systematic review. J Natl Cancer Inst.

[20] Baxter NN, Virnig DJ, Rothenberger DA, Morris AM, Jessurun J, Virnig BA (2005). Lymph node evaluation in colorectal cancer patients: a population-based study. J Natl Cancer Inst.

[21] Gonsalves WI, Kanuri S, Tashi T, Aldoss I, Sama A, AI-Howaidi I (2011). Clinicopathologic factors associated with lymph node retrieval in resectable colon cancer: A veteran’s affairs central cancer registry (VACCR) database analysis. J Surg Oncol.

[22] Leibl S, Tsybrovskyy O, Denk H (2003). How many lymph nodes are necessary to stage early and advanced adenocarcinoma of the sigmoid colon and upper rectum?. Virchows Arch.

[23] Tekkis P, Smith JJ, Heriot AG, Darzi AW, Thompson MR, Stamatakis JD (2006). A national study on lymph node retrieval in resectional surgery for colorectal cancer. Dis Colon Rectum.

[24] Chen HH, Chakravarty KD, Wang JY, Changchien CR, Tang R (2010). Pathological examination of 12 regional lymph nodes and long-term survival in stage I-III colon cancer patients: an analysis of 2,056 consecutive patients in two branches of same institution. Int J Colorectal Dis.

[25] Kelder W, Inberg B, Schaapveld M, Karrenbeld A, Grond J, Wiggers T (2009). Impact of the number of histologically examined lymph nodes on prognosis in colon cancer: a population-based study in the Netherlands. Dis Colon Rectum.

[26] Bilimoria KY, Stewart AK, Palis BE, Bentrem DJ, Talamoti MS, Ko CY (2008). Adequacy and importance of lymph node evaluation for colon cancer in the elderly. J Am Coll Surg.

[27] Vather R, Sammour T, Kahokehr A, Connolly AB, Hill AG (2009). Lymph node evaluation and long-term survival in stage II and stage III colon cancer: a national study. Ann Surg Oncol.

[28] Le Voyer TE, Sigurdson ER, Hanlon AL, Mayer RJ, Macdonald JS, Catalano PJ (2003). Colon cancer survival is associated with increasing number of lymph nodes analyzed: a secondary survey of intergroup trial INT-0089. J Clin Oncol.

[29] Feinstein AR, Sosin DM, Wells CK (1985). The Will Rogers phenomenon. Stage migration and new diagnostic techniques as a source of misleading statistics for survival in cancer. N Engl J Med.

[30] Eveno C, Nemeth J, Soliman H, Praz F, de The H, Valleur P (2010). Association between a high number of isolated lymph nodes in T1 to T4N0M0 colorectal cancer and the microsatellite instability phenotype. Arch Surg.

